# Validation of a water-load protocol to define the pattern of bladder sensation

**DOI:** 10.1007/s00192-018-3735-y

**Published:** 2018-08-18

**Authors:** Hayser Medina Lucena, Douglas G. Tincello

**Affiliations:** Department of Health Sciences, University of Leicester, Centre for Medicine, University Road, Leicester, Leicestershire LE1 7RH UK

**Keywords:** Urinary bladder, Sensation, Humans, Diuresis, Volunteers

## Abstract

**Introduction and hypothesis:**

The aim of this study was to confirm reliability of a water-load diuresis protocol and to assess the utility of bladder sensation curves.

**Methods:**

For confirmation of fixed diuresis rate (phase 1), 12 volunteers consumed 250–300 ml of water every 15 min and recorded bladder sensation on a visual analogue scale (VAS) every 5 min to maximum sensation over two filling cycles: voids 1 and 2 (V1 and V2). The test was performed twice. For test–retest validation (phase 2), 24 participants underwent the same protocol drinking 300 ml of water every 15 min. Diuresis rates and voided volumes were compared between cycles and across tests.

**Results:**

In phase 1, there was no difference in median void volume (V1 735 ml, V2 678 ml *p* = 0.433) or median diuresis rates (V2 12.1 ml/min, V3 14.4 ml/min *p* = 0.136) between cycles. When comparing those who drank 250–300 ml/15 min, there was less variability in those drinking 300-ml aliquots, so this was standardised for later experiments; 95% upper confidence limit of variability of the diuresis rate was calculated as 4.5 ml/min. Any test with a greater difference was rejected as invalid. In phase 2, only 16 participants were analysed. There was no difference in median void volumes between tests [V1 763 ml and 820 ml (*p* = 0.109) and V2 788 ml and 796 ml (*p* = 0.266)] or in diuresis rates between test 1 (12.33 ml/min) and 2 (14.40 ml/min) (*p* = 0.056). Median area under the curve was similar between test 1 404.96 and test 2 418.63.

**Conclusions:**

This refined protocol reliably produced stable diuresis with a water load of 300 ml/15 min, excluding those with a difference in diuresis rate > 4.5 ml/min.

## Introduction

Bladder sensation is a subjective complaint that is difficult to define, describe and measure. Sensation is defined as “a feeling, a translation into consciousness of the effects of stimuli exciting any of the organs of sense” [1]. In the case of bladder sensation, it corresponds to a sense of filling (physiological process), discomfort or pain (pathological) [2], where the nature of the stimulus is usually mechanical due to increased bladder volume, pressure or tension in the bladder wall [3], although inflammation and irritation produce similar sensations [4]. In other words, afferent noises such as pain stimuli, mechanical (stretch) stimuli and chemical release from the urothelium travel through different neuronal pathways; these signals inundate the central nervous system (CNS). However, it is unclear how the CNS decides which of these afferent noises is recognised and interpreted into bladder sensation [4].

Different methods have been described to measure bladder sensation, including cystometry [5–8], frequency volume charts [9], sensation-related bladder diaries (SR-BD) [10–13], visual analogue scales (VAS) [14, 15] and forced diuresis [16, 17]. There are conflicting data on which tool is the most accurate, especially as each method to evaluate bladder sensation may influence the way it is described by participants [7, 10, 11, 16, 18]. Most of these methods assess sensation by asking participants for three specific episodic events: first sensation of filling (FSF), first desire to void (FDV) and strong desire to void (SDV) [19]. These episodes are described as a separate event that does not correlate with the way bladder sensation develops, which is a constant and progressively increasing process [3].

Recently, there has been great interest in finding an objective way to measure bladder sensation using a non-invasive method [17, 18, 20]. The newly developed bladder sensation data-logging tool [21] is able to identify and record sensation during bladder filling [16]. This method uses a water-load protocol that potentially achieves a fixed diuresis rate [3, 17, 21, 22] and does not rely on the use of words, thus reducing observer bias. Individuals are asked to drink a large, constant amount of water to achieve a fixed diuresis rate and to record their bladder sensation on an X/Y graph while drinking [16, 17]. In recent work, we found the rate of diuresis measured across an entire filling cycle to have a high variability, in some cases >5 ml/min between tests in the same participant [16, 17] . These findings raised the question of whether the fluid-load protocol had been fully validated to confirm if constant diuresis had been achieved.

This study was designed to validate the original protocol [21] by calculating and comparing diuresis rates, to define the required volume to be consumed to achieve a constant diuresis rate, to confirm test–retest reliability and to graphically compare bladder sensation curves. Previous studies have looked at shapes of bladder sensation curves between healthy volunteers and patients with overactive bladder (OAB). They found that the intensity of the sensation in patients with OAB increases rapidly in comparison to volunteers [17, 23].

## Materials and methods

This was an observational and experimental study approved by West Midlands Research Ethics Committee. Volunteers were recruited by local advertisement within the university campus. Interested participants were given an information leaflet. Any volunteers diagnosed with any neurological disease, stroke, spinal injury, chronic kidney disease or cardiac disease were excluded. Following informed consent, a serum sample was obtained to measure glomerular filtration rate (GFR) to exclude undiagnosed kidney disease. The diuresis protocol was followed [21] except that volume aliquots to be drunk were adjusted based on hydration status derived from a biomedical impedance test performed immediately before starting, following advice from nephrology regarding the potential risk for water intoxication. Participants recorded bladder sensation on a 10-point VAS every 5 min while drinking an excess of water for the duration of the test. A bladder sensation data-logging tool recently developed and validated by McCarthy et al. was used [21]. It provides an easy approach to recording and quantifying sensations during filling and voiding [16].

Participants were asked to drink 250 ml of water every 15 min 1 h before the test. If they were over-hydrated on bio-impedance (+500 ml), they were asked to continue to drink water at the same rate during the test. If they were under-hydrated, this volume was increased to 300 ml/15 min. When they reached the strongest sensation they could bear, they were asked to hold on for up to 5 min and to remember this as their maximum sensation, marking it as a 10 on their sheet. They were then asked to void, and this volume was measured (V1). Immediately post-void, they recorded this as the minimal sensation.

Participants continued to drink and record bladder sensation for another entire filling cycle, the voided volume was measured (V2), and then again after at least 30 min into the next cycle (V3). The first cycle allowed participants to fix the maximum and minimum sensations in their minds; cycle two was the core part of the experiment on which analyses were done, and cycle three was used to confirm that a steady diuresis rate was achieved during cycles two and three (i.e. V2/t2 = V3/t3). This test was performed twice up to 14 days apart. The same instructions were read to participants at the beginning of each test.

Initially, 12 volunteers were asked to undergo the diuresis and bladder sensation protocol to confirm establishment of a fixed diuresis (phase 1), and then to collect a further 24 data sets to confirm test–retest reliability (phase 2). The aim was to confirm the reproducibility of voided volume between cycles and to compare diuresis rates within cycles of each test and between cycles. Following confirmation of reproducibility, we inspected sensation curves plotted from participants responses. In an attempt to quantify differences observed, the area under the curve (AUC) was calculated for each plot from the 24 data sets, and the median area for each cycle was compared as another test of reproducibility. Data were presented as median (range) and compared using Wilcoxon signed-rank test or Mann–Whitney *U* test for paired and unpaired data.

### Sample size and power calculations

Power calculation was not performed, as there are scarce data available on which to base the calculations. In essence, this is an exploratory and novel method to measure bladder sensation.

## Results

### Phase 1: Confirmation of fixed diuresis rate

Median age of the first 12 volunteers was 26 years (19–37), median body mass index (BMI) 29.1 kg/m^2^ (21.0–42.4). Nine were British, two Turkish and one Southeast Asian; 11 of 12 had the two tests within 14 days. One person withdrew after experiencing vomiting during the first test. All participants had a normal serum sodium concentration and GFR > 60 ml/min. On the first test ,11 of 12 had preloaded with 1000 ml (one drank 1250 ml). Seven drank 250 ml every 15 min and five drank 300 ml. There was no difference in V1 735 ml (386–1218 ml) and V2 678 ml (420–1064 ml) (*p* = 0.433). Median diuresis rates during V2/t2 and V3/t3 were 12.1 ml/min (8.94–17.18) and 14.4 (8.13–20.0), respectively (*p* = 0.136) (Table 1). Ranges of diuresis rates were wide. We compared the effect of different loading volumes (250 ml vs 300 ml) by calculating the difference in diuresis rates in cycles 2 and 3 and comparing them with volume loaded.

**Table 1:** Voids and diuresis rate at each test (phase 1)

Factor	Test 1 (*n* = 12)	Test 2 (*n* = 11)	*P* value**
V1 (ml) Median (range)	735 (386–1218)	618 (482–1042)	0.593
V2 (ml) Median (range)	678 (420–1064)	617 (490–1026)	0.929
*P* value*	0.433	0.533	
Diuresis rate V2 (ml/min) Median (range)	12.1 (8.94–17.18)	13.7 (8.90–16.36)	0.248
Diuresis rate V3 (ml/min) Median (range)	14.4 (8.13–20.0)	13.5 (9.16–16.33)	0.656
*P* value*	0.136	0.477	

*V1, V2 , V3* Voiding cycles 1, 2, and 3

*Comparison within each test, **comparison of Test 1 vs Test 2

**Fig. 1:**
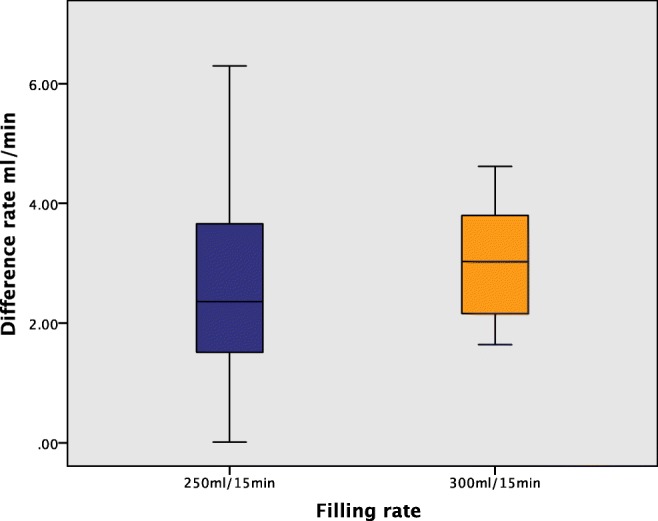
Median difference diuresis rate in Test 1

The median differences were 2.36 ml/min (0.01–6.30), with 250 ml/15 min and 3.03 ml/min (1.64–4.63) after 300 ml (*p* = 0.639). Although there was no statistically significant difference in the mean, the range of variation was much less with the larger-volume load (Fig. 1). We then performed the second test with participants all drinking 300 ml/15 min irrespective of hydration status.

During the second test, all participants had a preload of 1000 ml. Median V1 and V2 were 618 ml (482–1042 ml) and 617 ml (490–1026 ml), respectively (*p* = 0.533). Median diuresis rate of V2/t2 was 13.7 ml/min (8.90–16.36) and V3/t3 13.5 (9.16–16.33) (*p* = 0.477) (Table 1). The difference between diuresis rate in cycle 2 and cycle 3, where all participants drank 300 ml/15 min, was 0.53 ml/min (0.12–2.31). Analysis of data between the test cycles showed voided volumes and diuresis rate were not significantly different between tests (Table 1). Data for participants who drank 300 ml/15 min were analysed to identify the upper limit of variability of the diuresis rate between cycles. The upper limit of the 95% confidence interval (CI) of the mean difference of diuresis rate was 4.5 ml/min. Therefore, any value above this was rejected as invalid for the subsequent part of the study. Thus, we demonstrated that variability was minimised with a water load of 300 ml/15 min and determined that any test in which the variation in diuresis rate between the two test cycles exceeded 4.5 ml/min was to be rejected. Subsequently, 24 volunteers underwent the diuresis protocol with these changes to further validate the test.

### Phase 2: Confirmation of test–retest reliability

Twenty-five healthy volunteers were recruited; 24 underwent the forced diuresis protocol once and 21 twice. Three participants withdrew after the first test. Median age was 28 (19–47) years and median BMI 26.3 kg/m^2^ (19.0–39.0 kg/m^2^). Twenty-one were women and four were men. Five participants completed the two tests with a difference in diuresis rate > 4.5 ml/min and were excluded, leaving 16 for analysis, all of whom had a GFR > 60 ml/min and a normal serum sodium concentration. In the first test (Table 2), there was no difference in median voids: 763 ml (437–1052 ml) vs 788 ml (400–1136 ml) (*p* = 0.776). Median diuresis rate between cycle 2 and cycle 3 were the same [12.33 ml/min (8.43–17.38 ml/min) vs 12.01 ml/min (8.30–20.86 ml/min)] (*p* = 0.255). The median difference between cycle 2 and cycle 3 was 0.725 ml/min (0.08–2.88 ml/min).

**Table 2:** Voids and diuresis rate at each test (phase 2)

Factor	Test 1 (*n* = 16)	Test 2 (*n* = 16)	*P* value**
V1 (ml) Median (range)	763 (437–1052)	820 (474–1538)	0.109
V2 (ml) Median (range)	788 (400–1136)	796 (502–1489)	0.266
*P* value*	0.776	0.756	
Diuresis rate V2 (ml/min) Median (range)	12.33 (8.43–17.38)	14.40 (9.55–18.75)	0.056
Diuresis rate V3 (ml/min) Median (range)	12.01 (8.30–20.86)	13.28 (7.66–17.80)	0.326
*P* value*	0.255	0.215	

*V1, V2 , V3* Voiding cycles 1, 2, and 3

*Comparison of each test, **comparison of Test 1 vSs Test 2

**Fig. 2:**
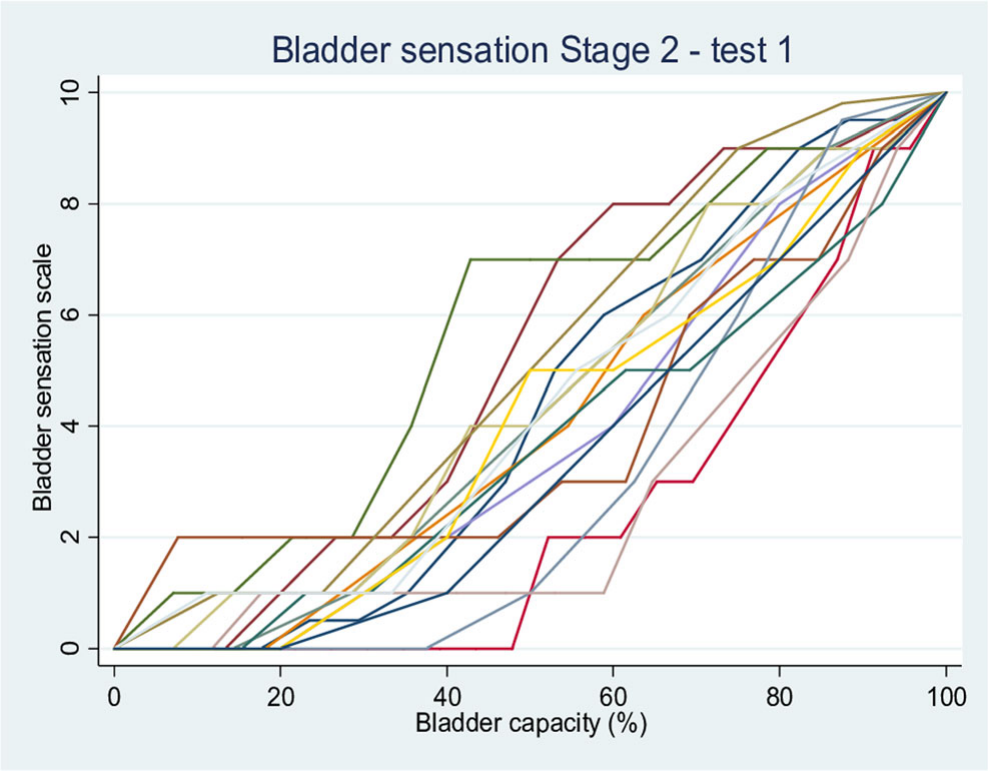
Bladder sensation pattern in Test 1 of the second phase of the study

**Fig. 3:**
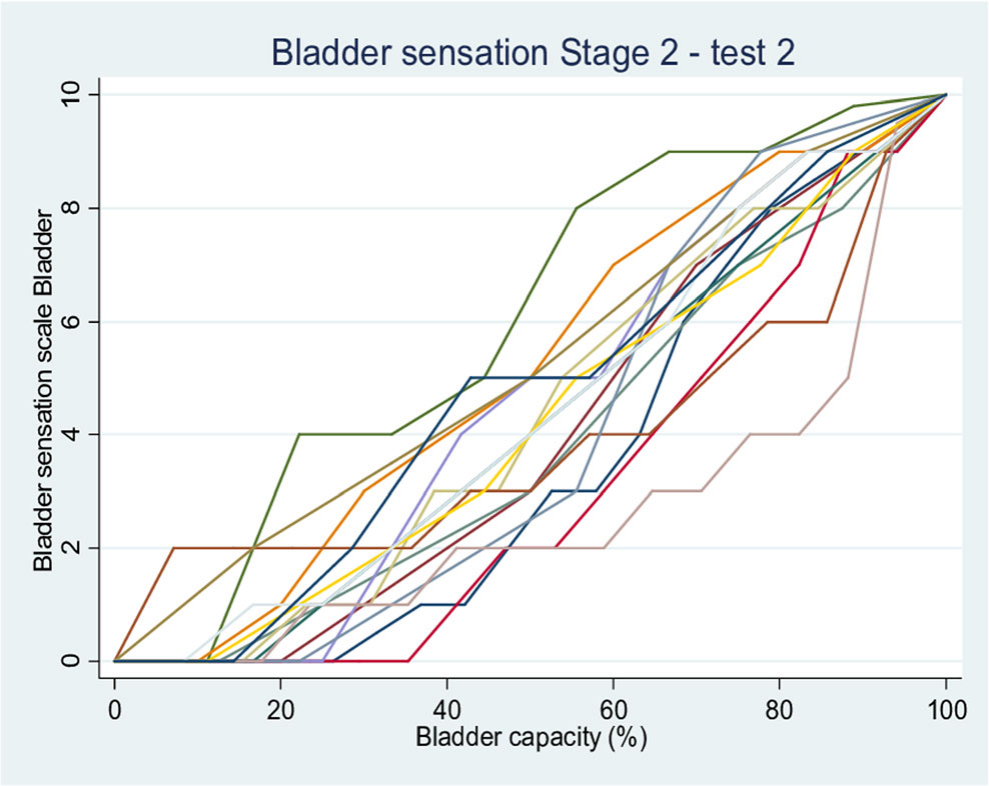
Bladder sensation pattern in Test 2 of second phase of the study

During the second test, there was no difference in median voids within or between cycles (Table 2). The difference in diuresis rate between cycles was 0.91 ml/min (0.08–3.46 ml/min). Again, test–retest comparisons showed no difference between voided volumes returned or the calculated diuresis rates (Table 2). Sensation from V2 are shown for the first Fig. 2) and second (Fig. 3) tests from the 16 complete data sets. Overall curves had a sigmoid appearance, with an initial slow rise in sensation, followed by an approximately linear increase, and then a plateau as bladder capacity was approached. There was variability in this pattern, with some volunteers returning a more linear curve and others a very marked sigmoid curve; a few had a distinct “step”. AUC was similar between tests: median 404.96 (247.28–557.14) in Test 1 vs 418.63 (262.50–596.02) in Test 2 (*p* = 0.234).

## Discussion

The data-logging tool described by McCarthy et al. [21] was novel and demonstrated the ability to graphically record bladder sensation. Bakali et al. [16] conducted a pilot study in women of different ethnic groups using the same protocol and found an increased variability in the mean diuresis rate between participants: 16 of 40 women had >5 ml/min differences [16]. This showed that further validation work needed to be undertaken. The forced diuresis protocol published by DeWachter et al. [17] was similar to the above protocol; they assessed diuresis rate by repeated voiding after 15-min intervals and confirmed this to be steady; data from volunteers showed all bladder sensation curves to be remarkably similar, with some degree of reproducibility.

It could be argued that testing for a steady rate of diuresis using 15-min repeated voids is not physiologically appropriate, since participants are required to void with no sensation; De Wachter et al. did not check for residual volumes during the test [17]. In our work, we measured diuresis rate across the whole filling cycle, which can be regarded as more physiologically accurate. This study answers the questions raised by Bakali et al. and proves that this protocol is both reliable and reproducible. However, this reproducibility is enhanced by using a fixed water intake of 300 ml every 15 min instead of 250 ml. We showed that maximum bladder sensation was achieved at similar bladder volumes within and between cycles. There was also no significant difference in diuresis rate between tests and within cycles.

The data-logging sheet measures and displays perception of bladder filling from an empty to a full bladder as well as in continuously, contemporaneously and noninvasively [23], rather than imposing arbitrary definitions of sensation, as is currently employed during cystometry. Although some could argue that the test is not a natural physiological process, as it forces diuresis, it is more physiological than cystometry [18, 22], which employs retrograde filling at high infusing rates and placement of urethral catheters, which both influence sensation [7, 24].

One of the first studies using this method only involved 11 healthy volunteers who underwent the forced diuresis protocol three times with a 10-day interval [17]. In contrast to the Bakali study [16], there was little variability in diuresis rate (12 ± 3 ml/min). It is unclear why variability in this study was less than in the Bakali et al. study when all participants drank at a similar rate.

When describing the pattern of sensation graphically, De Wachter et al. did a visual interpretation [17]. Two patterns were found (L- and S-shaped). Both curves had a similar start with a slow rise, which corresponded to a gradual increase in intensity. The second phase showed a steep rise in intensity: for the L-shape, it ended in absolute need to void; in the S-shape, there was a third phase, where again the sensation had a gradual increase before the need to void emerged. Despite the sample being too small to draw any significant conclusions, De Wachter’s study highlighted that this method could help healthcare personnel understand how the bladder sensation develops according to volume [17].

Two other studies have used focus groups in combination with the water-load protocol to assess bladder sensation [3, 23]. Both studies asked participants to go through three repetitive focus groups during which time they were asked to describe and locate their bladder sensation while undergoing the protocol. The purpose was to reach a consensus on the terms used to portray bladder sensation and to analyse bladder sensation curves. One study involved 11 healthy volunteers [3] and another ten patients with OAB [23]. Similar to the De Wachter et al. study [17], interpretation of bladder sensation curves were a visual conclusion. They found the curve in the patient group was steeper than for volunteers. Equally, there was little variability in diuresis rate. Interestingly, diuresis rates were lower in patients than in volunteers (6.9 ml ± 2.8 vs 12 ml ± 3, respectively). However, visual assessment of sensation curves is inevitably subjective, so we employed a mathematical interpretation of these complex curves to quantify the shape of the curve and add a degree of objectivity to the understanding of the progress and development of bladder sensation. It was reassuring that the median AUC for the two test cycles was the same, and we plan to exploit this mathematical approach to compare sensation curves between patients with different lower urinary tract symptoms (LUTS) in due course.

Some could argue that postvoid residual (PVR) should be have been measured to exclude any participants with urinary retention, although the main aim of this study was to validate a non-invasive test and describe how bladder sensation develops in daily life. It is also known that women >55 years, previous incontinence surgery and/or history of multiple sclerosis are predictors of increased PVR [25], and none of our participants met any of these parameters.

Now that this protocol has been validated, there is opportunity to use it, assess and explore the relationship between parameters of bladder sensation obtained in patients with different LUTS diagnoses. McCarthy et al. [21] provided purely descriptive information on the shape of the sensation curves presented. We applied a mathematical method to render the subjective evaluation of curves more reproducible and amenable to statistical comparisons, which confirms previous work by De Wachter et al. [17]. The water-load protocol achieves a constant high diuresis with rapid, non-invasive bladder filling. We refined and validated previous work [17, 21] and confirmed this is a feasible and optimal water-load protocol. We also defined what the expected range of within-test variability of diuresis rate should be.

## Abbreviations

CNS: Central nervous system
SR-BD: Sensation-related bladder diaries
VAS: Visual analogue scales
FSF: First sensation of filling
FDV: First desire to void
SDV: Strong desire to void
OAB: Overactive bladder
AUC: Area under the curve
BMI: Body mass index
LUTS: Lower urinary tract symptoms
PVR: Post-void residual

## References

[1] Stedman TL (2000). Stedman’s medical dictionary.

[2] Chapple CR, Artibani W, Cardozo LD, Castro-Diaz D, Craggs M, Haab F (2005). The role of urinary urgency and its measurement in the overactive bladder symptom syndrome: current concepts and future prospects. BJU Int.

[3] Heeringa R, de Wachter SGG, van Kerrebroeck PEV, van Koeveringe GA (2011). Normal bladder sensations in healthy volunteers: a focus group investigation. Neurourol Urodyn.

[4] Gillespie JI, Van Koeveringe GA, De Wachter SG, De Vente J (2009). On the origins of the sensory output from the bladder: the concept of afferent noise. BJU Int.

[5] Wyndaele JJ (1992). Are sensations perceived during bladder filling reproducible during cystometry?. Urol Int.

[6] Erdem E, Akbay E, Doruk E, Çayan S, Acar D, Ulusoy E (2004). How reliable are bladder perceptions during cystometry?. Neurourol Urodyn.

[7] De Wachter S, Van Meel TD, Wyndaele JJ (2008). Can a faked cystometry deceive patients in their perception of filling sensations? A study on the reliability of spontaneously reported cystometric filling sensations in patients with non-neurogenic lower urinary tract dysfunction. Neurourol Urodyn.

[8] Tsunoyama K, Sakakibara R, Takahashi O, Sugiyama M, Uchiyama T, Tateno F (2013). How the bladder senses? A five- grade measure. LUTS.

[9] Wachter SD, Wyndaele J (2003). Frequency-volume charts: a tool to evaluate bladder sensation. Neurourol Urodyn.

[10] Digesu GA, Basra R, Khullar V, Hendricken C, Camarata M, Kelleher C (2009). Bladder sensations during filling cystometry are different according to urodynamic diagnosis. Neurourol Urodyn.

[11] Naoemova I, Van Meel T, De Wachter S, Wyndaele J (2009). Does sensory bladder function during cystometry differ from that in daily life? A study in incontinent women. Neurourol Urodyn.

[12] Naoemova I, De Wachter S, Wyndaele J (2008). Comparison of sensation-related voiding patterns between continent and incontinent women: a study with a 3-day sensation-related bladder diary (SR-BD). Neurourol Urodyn.

[13] Naoemova I, De Wachter S, Wyts FL, Wyndaele JJ (2008). Reliability of the 24-h sensation-related bladder diary in women with urinary incontinence. Int Urogynecol J.

[14] Gallagher EJ, Bijur PE, Latimer C, Silver W (2002). Reliability and validity of a visual analog scale for acute abdominal pain in the ED. Am J Emerg Med.

[15] Dompeyre P, Fritel X, Bader G, Delmas V, Fauconnier A (2007). Bladder sensitivity testing using a visual analogue scale: comparative cystometric study on women. Neurourol Urodyn.

[16] Bakali E, Hong J, Gillespie J, Tincello D (2017). Saccharin increases perception of bladder filling in a forced diuresis experiment. Neurourol Urodyn.

[17] De Wachter SG, Heeringa R, Van Koeveringe GA, Winkens B, Van Kerrebroeck PE, Gillespie JI (2014). “Focused introspection” during naturally increased diuresis: description and repeatability of a method to study bladder sensation non-invasively. Neurourol Urodyn.

[18] De Wachter S, Smith P, Tannenbaum C, Van Koeveringe G, Drake M, Wyndaele JJ (2012). How should bladder sensation be measured?: ICI-RS 2011. Neurourol Urodyn.

[19] Abrams P, Cardozo L, Fall M, Griffiths D, Rosier P, Ulmsten U (2002). The standardisation of terminology of lower urinary tract function: report from the standardisation sub- committee of the international continence society. Obstet Gynecol.

[20] Nagle AS, Speich JE, De Wachter SG, Ghamarian PP, Le DM, Colhoun AF (2017). Non- invasive characterization of real- time bladder sensation using accelerated hydration and a novel sensation meter: an initial experience. (Report). Neurourol Urodyn.

[21] McCarthy A, Harvey J, Finney S, Gillespie J. Bladder awareness during filling and changes in sensation associated with the decision to void. (unpublished).

[22] Heeringa R, van Koeveringe GA, Winkens B, van Kerrebroeck PEV, de Wachter SGG (2011). Degree of urge, perception of bladder fullness and bladder volume—how are they related?. J Urol.

[23] Heeringa R, van Koeveringe GA, Winkens B, van Kerrebroeck PEV, de Wachter SGG (2012). Do patients with OAB experience bladder sensations in the same way as healthy volunteers? A focus group investigation. Neurourol Urodyn.

[24] Erdem E, Tunçkiran A, Acar D, Kanik EA, Akbay E, Ulusoy E (2005). Is catheter cause of subjectivity in sensations perceived during filling cystometry?. Urology.

[25] Milleman M, Langenstroer P, Guralnick ML (2004). Post- void residual urine volume in women with overactive bladder symptoms. J Urol.

